# Pharmacological regulation of protein-polymer hydrogel stiffness†

**DOI:** 10.1039/d3ra04046a

**Published:** 2023-08-15

**Authors:** Kun-Lin Wu, Ross C. Bretherton, Jennifer Davis, Cole A. DeForest

**Affiliations:** a Department of Chemical Engineering, University of Washington (UW) Seattle WA 98105 USA profcole@uw.edu; b Department of Bioengineering, UW Seattle WA 98105 USA; c Institute for Stem Cell & Regenerative Medicine, UW Seattle WA 98109 USA; d Center for Cardiovascular Biology, UW Seattle WA 98109 USA; e Department of Laboratory Medicine & Pathology, UW Seattle WA 98109 USA; f Department of Chemistry, UW Seattle WA 98105 USA; g Molecular Engineering & Sciences Institute, UW Seattle WA 98109 USA; h Institute for Protein Design, UW Seattle WA 98105 USA

## Abstract

The extracellular matrix (ECM) undergoes constant physiochemical change. User-programmable biomaterials afford exciting opportunities to study such dynamic processes *in vitro*. Herein, we introduce a protein-polymer hydrogel whose stiffness can be pharmacologically and reversibly regulated with conventional antibiotics. Specifically, a coumermycin-mediated homodimerization of gel-tethered DNA gyrase subunit B (GyrB) creates physical crosslinking and a rheological increase in hydrogel mechanics, while competitive displacement of coumermycin with novobiocin returns the material to its softened state. These unique platforms could potentially be modulated *in vivo* and are expected to prove useful in elucidating the effects of ECM-presented mechanical signals on cell function.

## Abstract 2

Hydrogel biomaterials partially modified with GyrB can be stiffened/softened *via* small molecule pharmacological-mediated protein (de)dimerization.

## Graphical abstract

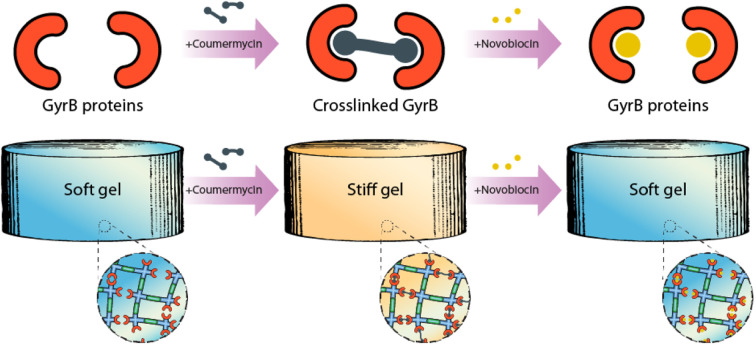

The extracellular matrix (ECM) scaffolds all tissues and organs, serving as an important non-cellular component that supports cellular structure. ECM also directs cell fate and function, including migration, proliferation, and differentiation, providing spatiotemporally varied biophysical and biochemical signaling cues to embedded cells.^1^ Such cues draw out biochemical responses to regulate gene transcription and therefore change cell shape and cytoskeletal dynamics to govern cell growth as well as cell–cell communication.^2,3^ Human stem cells can specify their lineage in response to varied substrate elasticity,^4^ just as ECM stiffness can modulate proliferation through stimulated cyclin D1-dependent G1 cell cycle progression.^3^ These observations demonstrate that ECM-presented biophysical signals, especially material stiffness, play a critical role in regulating cell fate.^5^

Despite this existing knowledge, understanding the specific role of ECM on cell function remains a challenge to achieve *in vivo*. The topology and composition of ECM is not only unique to each tissue, but the ECM structure is also highly dynamic. For example, bone tissue has an average larger elastic modulus – a common measurement for stiffness – than brain tissue so that it can provide structural protection for organs.^6^ Moreover, cells exhibit different mechanobiological responses depending on their mechanical dosing, exhibiting a so-called “mechanical memory”.^7^ As such, it is desirable to develop cell culture platforms where individual parameters can be systematically tuned, particularly those whose biochemical and biophysical properties can be reversibly manipulated on demand. Researchers have turned to simplified biomaterials to further their understanding and hydrogels have proven to be a particularly attractive class of materials. Critical characteristics of hydrogels, including their high-water content, tissue-like elasticity, and facile transport of nutrients and waste, recapitulate critical aspects of the native ECM. In addition, cytocompatible hydrogel formulations can be employed to support cell encapsulation.^8^ The initial stiffness and degradability of hydrogels can be simply modulated through a variety of chemical approaches, making them an ideal candidate to mimic ECM and be applied in complex cell culture environments.

Many strategies exist to form hydrogels with different static mechanics; however, far fewer exist to modify mechanical properties on demand in ways that recapitulate many developmental processes, homeostasis, and disease progression. Efforts to probe the biophysical responses to ECM that can either soften or stiffen over time are needed. Stimuli-responsive cell culture platforms are a powerful tool to recapitulate *in vivo* cellular environments, owing to their capabilities to respond external stimuli with temporal and spatial precision.^9,10^ Several techniques to date have demonstrated that cell fate can be controlled through stiffness mediation with light,^11–14^ voltage,^15^ pH,^16^ and temperature.^17,18^

Deviating from conventional external triggers for material modulation, we sought to exploit an input that could be theoretically administered intravenously, opening the door to *in vivo* material modulation. Towards this, we were inspired by recent approaches from the Weber lab, demonstrating pharmacological-triggered material degradation could be used for controlled drug release from hydrogel depots.^19–21^ Complementing constructs that undergo complete dissolution following small-molecule treatment, our goal was to extend these methods to create materials capable of controlled reversible stiffening. To do so, we sought to create step-growth hydrogels that were partially modified with GyrB, such that small molecule pharmacological-mediated protein (de)dimerization which could be used to control material stiffening and softening (Fig. 1). Since both coumermycin and novobiocin bind to the amino-terminal subdomain of GyrB with high affinity (*K*_d_, 10^−8^ M),^22^ but with different valencies (*i.e.*, coumermycin binds two GyrB, novobiocin only one), we hypothesized that this mechanism could be exploited for inducible physical hydrogel (un)crosslinking.

**Fig. 1:**
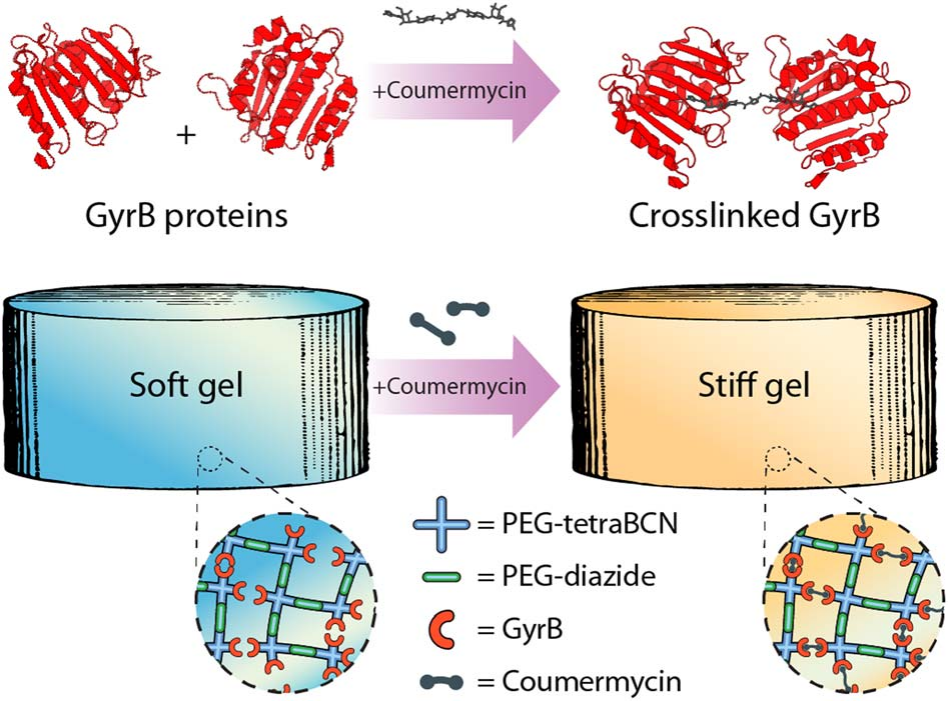
Coumermycin-mediated homodimerization of GyrB can be exploited for pharmacological crosslinking of hydrogel biomaterials.

Intent on using the bioorthogonal strain-promoted azide–alkyne cycloaddition (SPAAC) to form hydrogels, we first expressed and purified GyrB containing a single azide handle (Fig. 2). For this, we exploited the sortase-tag enhanced protein ligation (STEPL) method,^23–27^ recombinantly expressing GyrB in *E. coli* as a fusion with the sortase recognition sequence (LPETG), a flexible Gly–Ser spacer, *S. aureus* sortase A (SrtA), and a polyhistidine tag. In the presence of calcium and an azide-containing polyglycine probe [H-GGGGDDK(N_3_)-NH_2_, ESI Methods S1 and S2],† an intramolecular sortase-mediated transpeptidation ligates the azide-containing species to GyrB while cleaving the polyhistidine-tagged SrtA. If transpeptidation is initiated during immobilized metal affinity chromatography, the cleaved SrtA will remain bound to the Ni-NTA column, permitting the C-terminally azide-tagged GyrB-N_3_ to be generated and purified in a single step (Fig. 2a). Sodium dodecyl sulfate-polyacrylamide gel electrophoresis (SDS-PAGE) and whole-protein mass spectrometric analyses indicate that the monotagged product was obtained by STEPL in good purity and with the correct mass (Fig. 2b, ESI Fig. S1, ESI Method S3 and S4).†

**Fig. 2:**
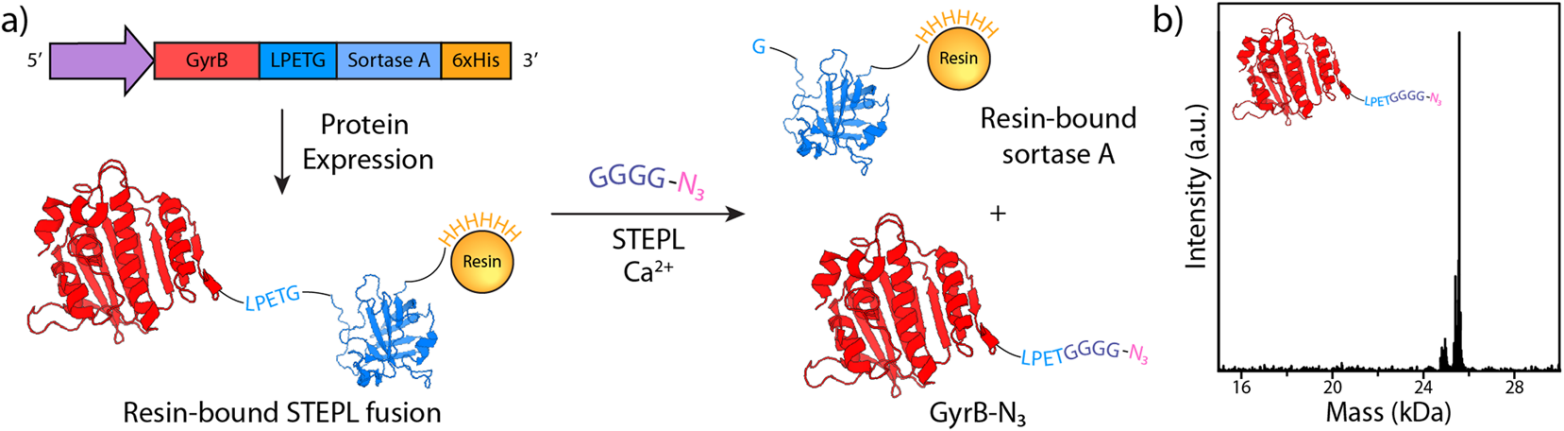
Chemoenzymatic synthesis and validation of GyrB-N_3_. (a) STEPL permits one-step protein purification and C-terminal monofunctionalization of GyrB with a clickable azide handle. GyrB is appended with the genetically encoded sorting signal (LPETG) are expressed as a fusion with sortase A and a polyhistidine tag. Following chromatographic isolation, a calcium-catalyzed transpeptidation of an azide-modified polyglycine probe promotes ligation and concomitant displacement from the polyhistidine-tagged sortase. (b) Whole-protein mass spectrometry indicates that the desired GyrB-N_3_ product is recovered by STEPL.

With GyrB-N_3_ in hand, we created hydrogels through SPAAC reaction^28,29^ of a four-arm poly(ethylene glycol) (PEG) bicyclononyne (PEG-tetraBCN, 3 mM, *M*_n_ ∼ 20 kDa), a linear PEG-diazide (4.65 mM, *M*_n_ ∼ 3.4 kDa), and GyrB-N_3_ (0, 1.35, or 2.7 mM) in phosphate-buffered saline (Fig. 3a and b). Hydrogels were formed both with and without GyrB-dimerizing coumermycin (0 or 1.35 mM, preincubated with GyrB-N_3_ for 1 hour prior to combination with other gel precursors) and analysed *via in situ* rheometry (ESI Method S5, ESI Fig. S2†). Gels formed at similar rates, reaching complete gelation in ∼1 h, but were significantly stiffer when both GyrB and coumermycin were included (Fig. 3c). Moreover, the extent of hydrogel stiffening was observed to scale with GyrB concentration when coumermycin was present, but not in its absence, indicating that coumermycin could successfully crosslink these protein-polymer hydrogels (Fig. 3d). Experiments in which preformed hydrogels were incubated in a coumermycin-containing solution prior to mechanical testing were also performed, with results trending similarly with those shown in Fig. 3d (in which coumermycin was included during hydrogel formation). These efforts were largely abandoned, however, due to challenges in reproducibly loading/analyzing precast hydrogels on the rheometer.

**Fig. 3:**
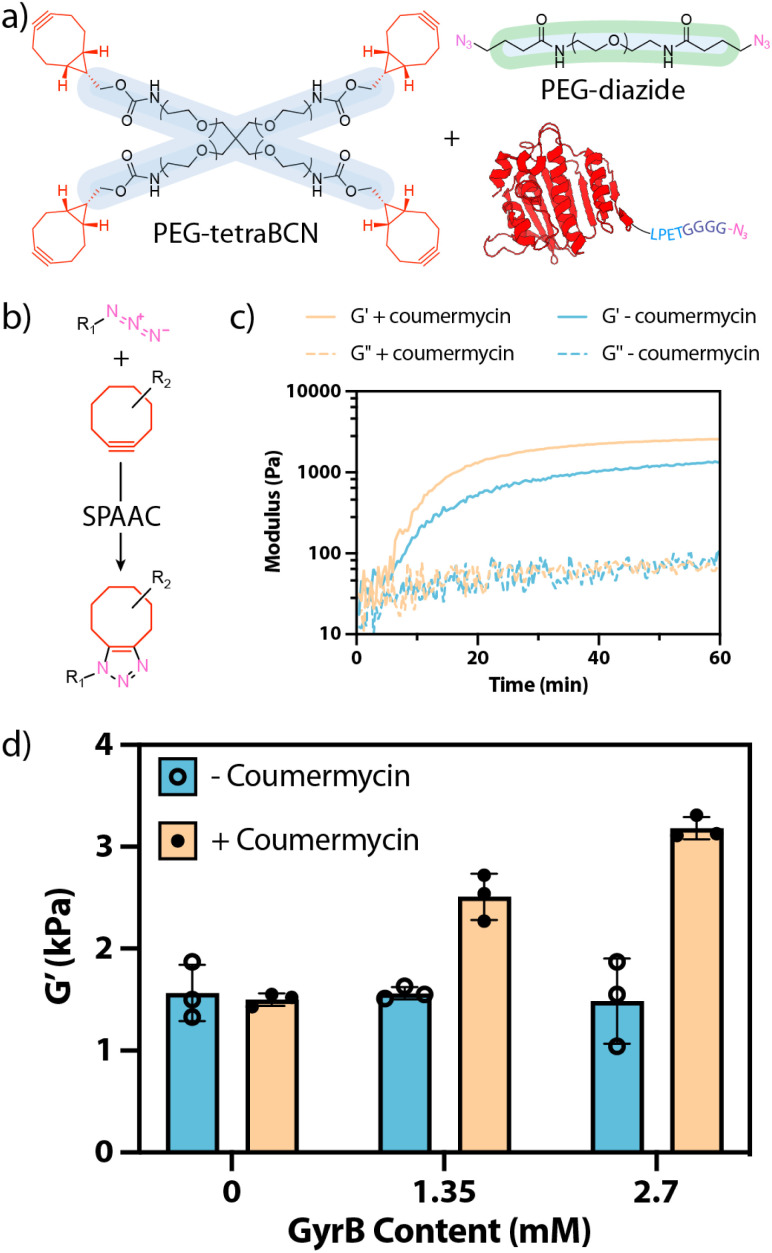
(a) Macromolecular precursors are polymerized *via* step-growth polymerization into bulk hydrogels. (b) Gels form *via* SPAAC reaction between reactive azides and ring-strained BCN moieties. (c) *In situ* rheometry indicates that GyrB-containing SPAAC hydrogels including coumermycin form at a similar rate but with higher stiffness than those that do not. (d) Hydrogel stiffness, as measured by *in situ* rheometry, scales with GyrB concentration, but only when coumermycin is present.

Having demonstrated that coumermycin could be used to create comparatively stiff GyrB-containing biohybrid gels through secondary physical crosslinking, we turned our attention towards controlling subsequent material softening through novobiocin's competitive displacement of coumermycin (Fig. 4a). Here, material stiffness of gels formed with(out) coumermycin (0 or 1.35 mM), as well as with(out) novobiocin (0 or 13.5 mM), was assessed rheometricly (Fig. 4b). Coumermycin dimerization of GyrB-N_3_ was allowed to proceed for 1 hour prior to addition of novobiocin (1 hour), both before addition of PEG-tetraBCN and PEG-diazide. In gels lacking coumermycin, inclusion of novobiocin yielded no decrease in moduli, as expected. Gels containing coumermycin alone were significantly stiffer than those without, while those supplemented with novobiocin softened to the same moduli as gels lacking coumermycin.

**Fig. 4:**
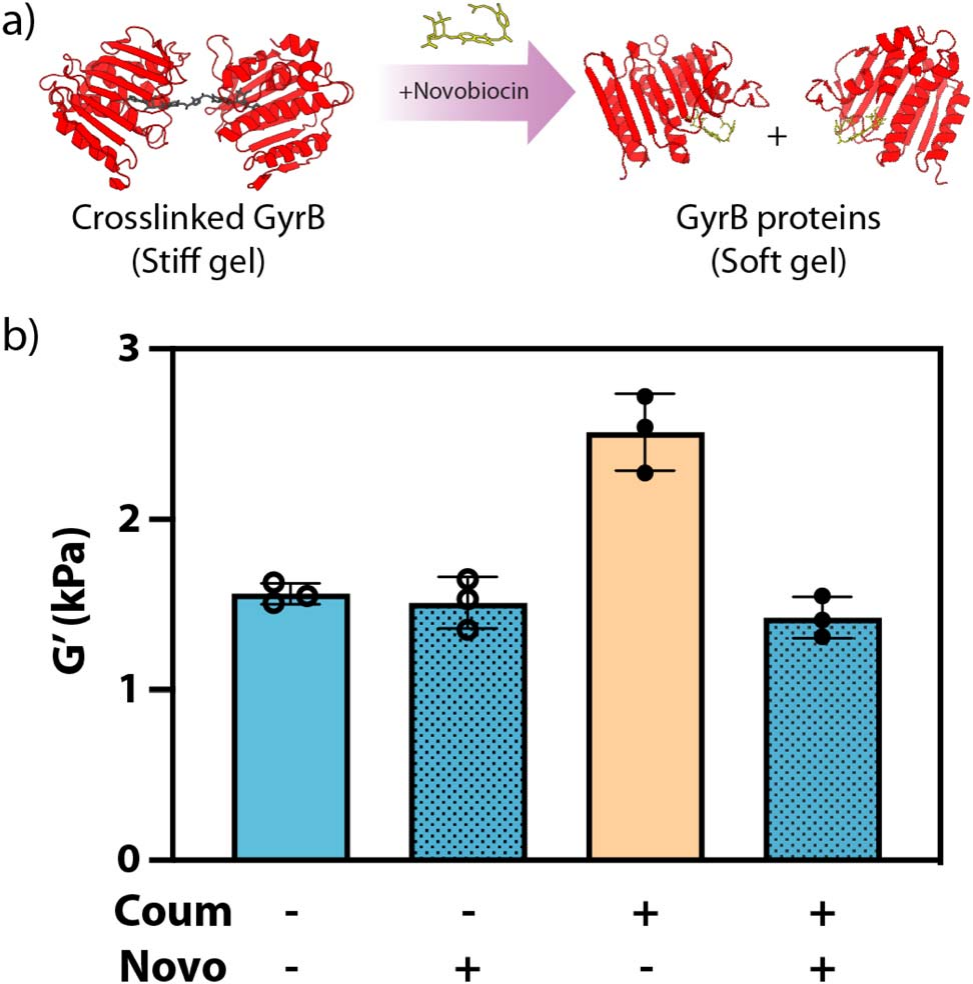
(a) Competitive displacement of coumermycin with novobiocin enables pharmacological softening of GyrB SPAAC hydrogels. (b) Hydrogels containing both coumermycin (coum) and novobiocin (novo) exhibit similar stiffnesses with those lacking coumermycin, as measured by *in situ* rheometry.

Our findings illustrate how the stiffness of biomaterials can be pharmacologically modified through controlled protein dimerization. Drug-responsive biohybrid hydrogels were synthesized through SPAAC click chemistry to incorporate PEG polymers with GyrB fusion proteins, allowing common aminocoumarin antibiotics to dimerize in between. Rheological studies of these novel materials were conducted to investigate the reversible stiffness changes through the presence or absence of coumermycin/novobiocin antibiotics. While such studies extend beyond the scope of this initial communication, we anticipate that this material strategy could uniquely enable regulation of material stiffness in the presence of living cells and potentially *in vivo*, strategies that could yield new approaches for tissue engineering and drug delivery.

## Conflicts of interest

There are no conflicts to declare.

## Supplementary Material

RA-013-D3RA04046A-s001
