# Supplementary material for: Pharmacological regulation of protein-polymer hydrogel stiffness
Source: RSC Adv. 2023 Aug 15;13(35):24487–90. doi: 10.1039/d3ra04046a (PMC10426327; doi:10.1039/d3ra04046a)
Supplement: RA-013-D3RA04046A-s001 [file RA-013-D3RA04046A-s001.pdf]

Electronic Supplementary Material (ESI) for RSC Advances.
This journal is © The Royal Society of Chemistry 2023

Supplementary Information for

Pharmacological Regulation of Protein-Polymer Hydrogel Stiffness

Kun-Lin Wu, Ross C. Bretherton, Jennifer Davis, Cole A. DeForest*

*Corresponding author. Email: profcole@uw.edu

Table of Contents

General synthetic information........ 3

Synthesis of previously reported compounds used in this work........ 4

Method S1 Fmoc solid-phase peptide synthesis........ 5

Method S2 Synthesis of H-GGGGDDK(N_3)-NH_2........ 6

Method S3 Construction of GyrB STEPL plasmid........ 7

Method S4 GyrB-N_3 expression and purification by STEPL........ 8

Figure S1 STEPL purification of GyrB-N_3........ 9

Method S5 In situ rheology of hydrogel formation........ 10

Figure S2 Strain and frequency sweep analysis of hydrogels........ 11

References........ 12

General synthetic information

Chemical reagents and solvents were purchased from either Sigma-Aldrich or Fisher Scientific and used as received unless otherwise noted. Peptide synthesis reagents were purchased from either ChemPep or Chem-Impex and used as received. Deionized water (dH_2O) was generated by a U.S. Filter Corporation Reverse Osmosis System with a Desal membrane. Synthetic chemical reactions were performed under a nitrogen atmosphere in oven-dried glassware and stirred with a Teflon-coated magnetic stir bar unless otherwise noted. Solvents were removed *in vacuo* with a Büchi Rotovapor R-3 equipped with a V-700 vacuum pump and V-855 vacuum controller and a Welch 1400 DuoSeal Belt-Drive high vacuum pump. ^{1}H nuclear magnetic resonance (NMR) data was collected at 298 K on Bruker instruments and chemical shifts are reported relative to tetramethylsilane (TMS, $\delta = 0$). Microwave-assisted peptide synthesis was performed on a CEM Liberty 1. Semi-preparative reversed-phase high-pressure liquid chromatography (RP-HPLC) was performed on a Dionex Ultimate 3000 equipped with a variable multiple wavelength detector, automated fraction collector, and Thermo 5 µm Synchronis silica 250 x 21.2 mm C18 column. Lyophilization was performed on a LABCONCO FreeZone 2.5 Plus freeze-dryer equipped with a LABCONCO rotary vane 117 vacuum pump. High-resolution mass spectrometry (HRMS) was performed on a Thermo Linear Trap Quadrupole Orbitrap Xcalibur 2.0 DS. Whole-protein mass spectrometry was performed using a Waters Synapt – G2 QTOF. Rheological measurements were performed on an Anton Paar MCR301 equipped with a C-PTD200 Peltier plate and a CP25-1 cone and plate geometry.

Synthesis of previously reported compounds used in this work

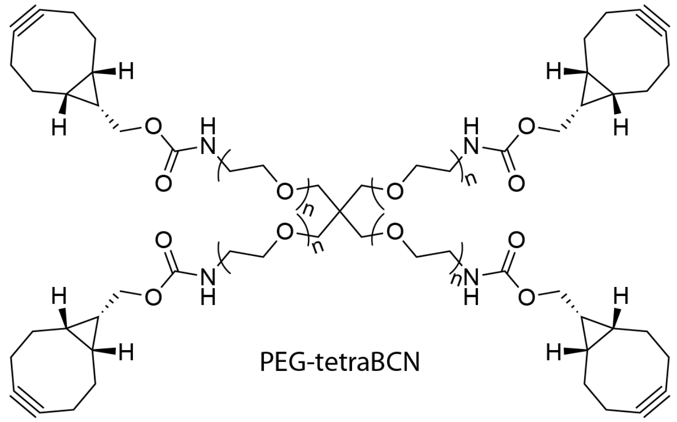

PEG-tetraBCN

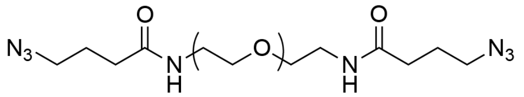

PEG-diazide

Poly(ethylene glycol) tetrabicyclononyne (PEG-tetraBCN, M_n ~ 20,000 Da) and poly(ethylene glycol) diazide (PEG-diazide, M_n ~ 3,400 Da) were synthesized as previously reported[1,2].

Method S1 Fmoc solid-phase peptide synthesis

A CEM Liberty 1 was used to perform microwave-assisted Fmoc solid phase peptide synthesis (SPPS, 0.25 mmol scale). Fmoc deprotection was performed in 20% piperidine (v/v) in dimethylformamide (DMF) with 1-hydroxybenzotriazole (HOBt, 0.1 M, 90 °C, 90 sec). Amino acids were coupled to resin-bound peptides upon treatment (75 °C, 5 min) with Fmoc-protected amino acid (2 mmol, 4x), 2-(1H-benzotriazol-1-yl)-1,1,3,3-tetramethyluronium hexafluorophosphate (HBTU, 2 mmol, 4x), and *N*,*N*-diisopropylethylamine (DIEA, 2 mmol, 4x) in a mixture of DMF (9 mL) and *N*-Methyl-2-pyrrolidone (NMP, 2 mL). Room-temperature couplings were performed without microwave assistance (1 hr).

Method S2 Synthesis of H-GGGGDDK(N_3)-NH_2

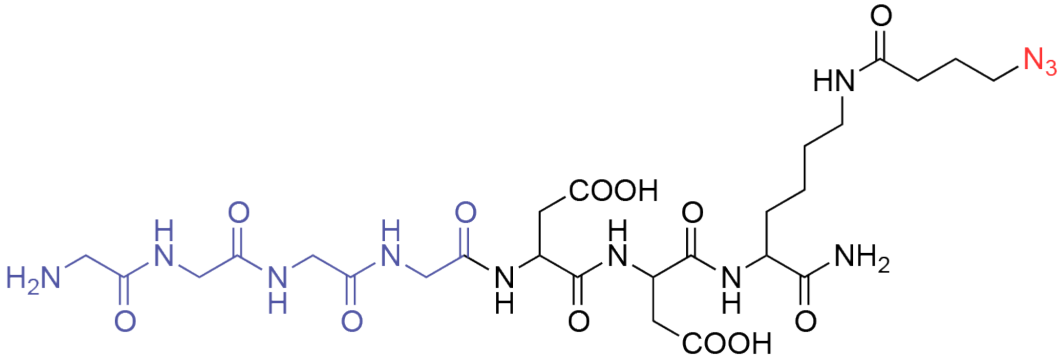

The resin-bound peptide Boc-GGGGDDK(Mtt)-NH_2 was synthesized by Fmoc SPPS (Method S1) on Rink amide resin (0.25 mmol scale). The resin was washed with DMF (3x) and dichloromethane (DCM, 3x) prior to Mtt cleavage [2 min, 15 mL, 97:2:1 DCM:Triisopropylsilane (TIS):Trifluoroacetic Acid (TFA), 9x]. Resin was washed (DCM, 3x; DMF, 3x) prior to treatment (1 hr) with N_3-COOH (4x, 1 mmol, 129 mg) by HATU coupling (3.95x, 0.988 mmol, 188 mg) and N,N-Diisopropylethylamine (DIEA, 8x, 2 mmol, 174 µL) in minimal DMF. Resin was washed (DMF, 3x; DCM, 3x) prior to peptide cleavage/deprotection (95:5 TFA:H_2O, 20 mL, 2 hr) and precipitation (diethyl ether, 180 mL, 0 °C, 2x). The crude peptide was purified *via* RP-HPLC using a 55-minute gradient from 5-100% acetonitrile:H_2O; lyophilization yielded the final product (H-GGGGDDK(N_3)-NH_2) as a white solid (62 mg, 0.087 mmol, 34% overall yield). Peptide purity was confirmed using MALDI-TOF: calculated for $C_{26}H_{43}N_{12}O_{12}^+$ [M + ^{1}H]$^+$, 715.31; observed 715.08.

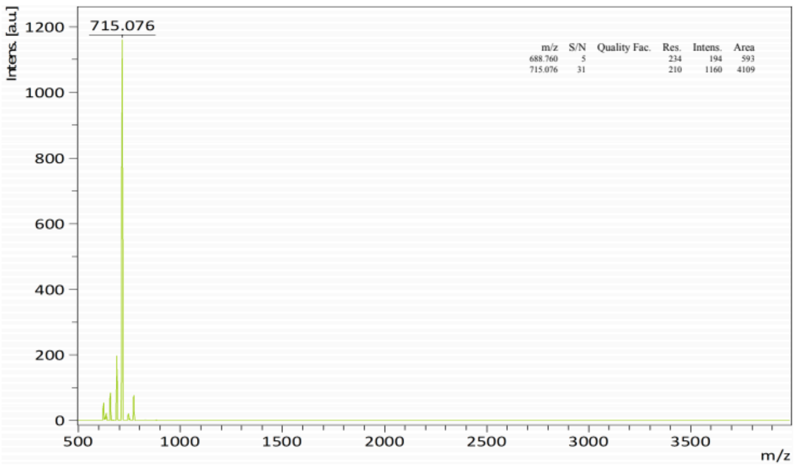

Method S3 Construction of GyrB STEPL plasmid

The GyrB gene with two restriction enzyme sites (i.e., NdeI and XhoI) at each end was obtained in the form of gBlocks through Integrated DNA Technologies (IDT). Both the GyrB gene and the STEPL plasmid template[2] underwent restriction digest using NdeI and HindIII restriction enzymes and were then ligated together by applying T4 DNA Ligase. The newly constructed plasmid was transformed into Rubidium Chloride (RbCl)-competent Top 10 E. *Coli* and plated onto agar plates (5 g Yeast Extract, 10 g NaCl, 10 g Tryptone, 15 g Agar, 1 L dH_2O) containing carbenicillin (100 µg/mL). Colonies were formed on the plates and selected through antibiotic resistance. Plasmid mini preparation was then performed to extract and purify the plasmids. The sequence was confirmed by sending the resulting product to GENEWIZ for verifying correct mutation.

Sequence-confirmed GyrB STEPL plasmid was transformed into chemically competent BL21 cells for protein expression. The cells were plated onto agar plates (5 g Yeast Extract, 10 g NaCl, 10 g Tryptone, 15 g Agar, 1 L dH_2O) containing carbenicillin (100 µg/mL). Successful colonies were selected for expression.

GyrB sequence was fused with LPXTG motif, GGS linked and STEPL sequence to undergo sortase expressed protein ligation for protein synthesis. The DNA sequences of the mutant were shown below reading from 5' end to 3' end. The GyrB sequences, LPETG, GGS linker, and STEPL were marked black, blue, green, and red for recognition.

GyrB-LPETG-$(GGS)_5$-Sortase A-6xHis:
ATGTGCTCGAATTCTTATGACTCCTCCAGTATCAAAGTCCTGAAAGGGCTGGATGCGGTGCGTA
AGCGCCCGGGTATGTATATCGGCGACACGGATGACGGCACCGGTCTGCACCACATGGTATTCGA
GGTGGTAGATAACGCTATCGACGAAGCGCTCGCGGGTCACTGTAAAGAAATTATCGTCACCATT
CACGCCGATAACTCTGTCTCTGTACAGGATGACGGGCGCGGCATTCCGACCGGTATTCACCCGG
AAGAGGGCGTATCGGCGGCGGAAGTGATCATGACCGTTCTGCACGCAGGCGGTAAATTTGACGA
TAACTCCTATAAAGTGTCCGGCGGTCTGCACGGCGTTGGTGTTTCGGTAGTAAACGCCCTGTCG
CAAAAACTGGAGCTGGTTATCCAGCGCGAGGGTAAAATTCACCGTCAGATCTACGAACACGGTG
TACCGCAGGCCCCGCTGGCGGTTACCGGCGAGACTGAAAAAACCGGCACCATGGTGCGTTTCTG
GCCCAGCCTCGAAACCTTCACCAATGTGACCGAGTTCGAATATGAAATTCTGGCGAAACGTCTG
CGTGAGTTGTCGTTCCTCAACTCCGGCGTTTCCATTCGTCTGCGCGACAAGCGCGACGGCAAAG
AAGACCACTTCCACTATGAAGGCCTCGAGCTGCCGGAAACCGGTGGTGGTAGTGGTGGCTCTGG
CGGTTCTGGTGGCAGTGGCGGTAGCCAAGCTAAACCTCAAATTCCGAAAGATAAATCAAAAGTG
GCAGGCTATATTGAAATTCCAGATGCTGATATTAAAGAACCAGTATATCCAGGACCAGCAACAC
CTGAACAATTAAATAGAGGTGTAAGCTTTGCAGAAGAAAATGAATCACTAGATGATCAAAATAT
TTCAATTGCAGGACACACTTTCATTGACCGTCCGAACTATCAATTTACAAATCTTAAAGCAGCC
AAAAAAGGTAGTATGGTGTACTTTAAAGTTGGTAATGAAACACGTAAGTATAAAATGACAAGTA
TAAGAGATGTTAAGCCAACAGATGTAGAAGTTCTAGATGAACAAAAAGGTAAAGATAAACAATT
AACATTAATTACTTGTGATGATTACAATGAAAAGACAGGCGTTTGGGAAAAACGTAAAATCTTT
GTAGCTACAGAAGTCAAACATCACCACCATCATCACTAA

Method S4 GyrB-N_3 expression and purification by STEPL

LB (500 mL) supplemented with ampicillin (100 µg mL^{-1}) was inoculated with an overnight cell culture (10 mL) and incubated (37 °C) with agitation (250 rev min^{-1}). After reaching an optical density at λ = 600 nm of 0.6, isopropyl β-D-1-thiogalactopyranoside was added (final concentration of 0.5 mM) and expression was continued overnight under reduced temperature (18 °C).

Cells were harvested *via* centrifugation (7,000 g, 10 min). The cell pellet was resuspended in lysis buffer (40 mL, 20 mM Tris, 50 mM NaCl, 10 mM imidazole, 1 mM phenylmethylsulfonyl fluoride) and sonicated on ice (6 cycles of 3 minutes at 30% amplitude 33% duty cycle and 3 min resting). Soluble and insoluble fractions were separated *via* centrifugation (5,000 g, 20 min).

Clarified lysate was applied to Ni-NTA resin (2.5 mL) and incubated under mild agitation (4 °C, 1 hr). The flow-through was discarded, and the resin was washed with wash buffer (20 mM Tris, 50 mM NaCl, 20 mM imidazole, 20 mL, 5x) and STEPL buffer (20 mM Tris, 50 mM NaCl, 20 mL, 1x). H-GGGGDDK(N_3)-NH_2 (20 molar excess) was added to resin in conjugation buffer (20 mM Tris, 50 mM NaCl, 100 µM $CaCl_2$, 2 mL) to promote intramolecular sortagging (37 °C, 4 hr).

The conjugated protein solution was collected, and the resin was washed with STEPL buffer (1 mL, 5x) to collect any remaining protein. The protein solution was dialyzed against STEPL buffer using ThermoFisher SnakeSkin Dialysis Tubing (molecular weight cut-off, MWCO ~ 10 kDa) to remove any unconjugated peptide and concentrated using an Amicon centrifugal spin column (MWCO ~ 10 kDa). Typical yields for purified GyrB-N_3 following STEPL were ~10 mg per liter of cell culture.

Figure S1 STEPL purification of GyrB-N_3

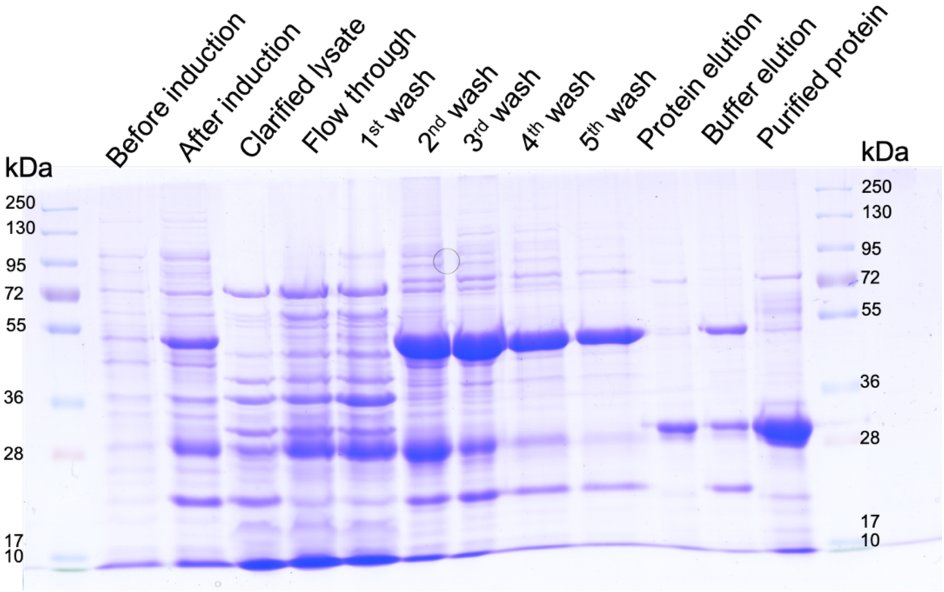

GyrB-N_3 is generated through STEPL involving GyrB and H-GGGGDDK(N_3)-NH_2 peptide. The GyrB-SrtA-6xHis fusion appears as a ~50 kDa band post induction. After chromatographic immobilization on Ni-NTA, the resin is incubated with the polyglycine probe; the sortagged protein is collected in the eluent, while the SrtA-6xHis protein remains column-bound. Subsequent resin washes with imidazole displaces the SrtA-6xHis and regenerates the Ni-NTA. The peptide-eluted sortagged protein (GyrB-N_3) is a pure (>95%) homogenous species appearing as a band ~29 kDa.

Method S5 In situ rheology of hydrogel formation

GyrB-N_3 (0, 1.35, or 2.7 mM) in PBS was preincubated with coumermycin (0 or 1.35 mM) for 1 hr prior to further incubation with novobiocin (0 or 13.5 mM) for 1 hr (final in-gel concentrations given).

PEG-tetraBCN (3 mM) was added to a solution of PEG-diazide (4.65 mM) and antibiotic-treated GyrB-N_3 (0, 1.35, or 2.7 mM) in PBS (final concentrations given). Upon mixing, precursors (30 μL) were immediately transferred into a parallel-plate geometry (8 mm diameter, 0.5 cm spacing) of an Anton Paar MCR301 instrument for in situ oscillatory rheology studies. The storage and loss moduli (G' and G" respectively) were measured at constant strain (1%) and frequency (1 rad/s), conditions determined to fall within the linear viscoelastic region (Figure S2).

Figure S2 Strain and frequency sweep analysis of hydrogels

PEG-tetraBCN (3 mM) was added to a solution of PEG-diazide (4.65 mM) and GyrB-N_3 (1.35 mM) in PBS (final concentrations given). Upon mixing, precursors (30 µL) were immediately transferred into a parallel-plate geometry (8 mm diameter, 0.5 cm spacing) of an Anton Paar MCR301 instrument for in situ oscillatory rheology studies. Strain sweeps were conducted between 1 – 100% strain at constant frequency (1 rad/s). Frequency sweeps were conducted between 10^{-3} – 10^{2} rad/s at constant strain (1%).

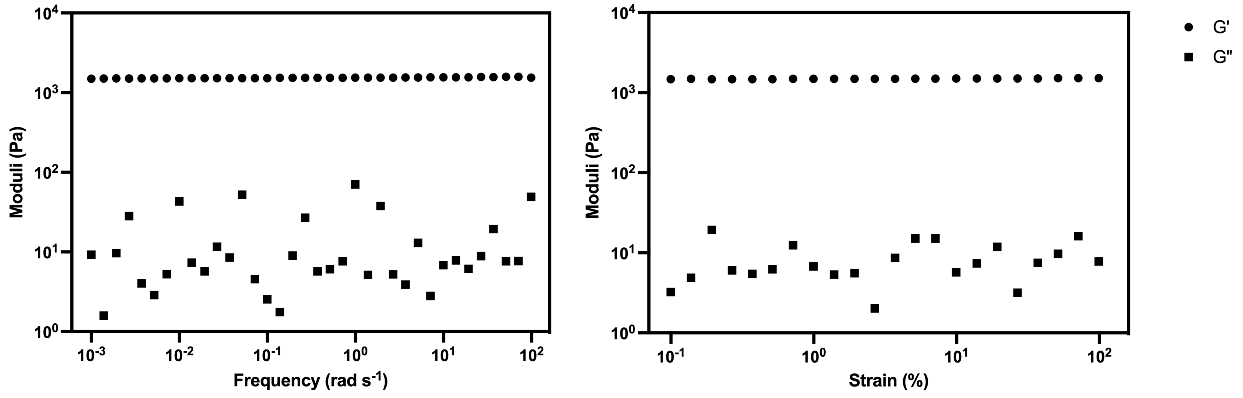

References

1 C. A. DeForest and D. A. Tirrell, *Nature Materials*, 2015, **14**, 523–531.
2 J. A. Shadish, G. M. Benuska and C. A. DeForest, *Nature Materials*, 2019, **18**, 1005–1014.
